# Phosphorylated Mammalian Target of Rapamycin p-mTOR Is a Favorable Prognostic Factor than mTOR in Gastric Cancer

**DOI:** 10.1371/journal.pone.0168085

**Published:** 2016-12-22

**Authors:** Guo-dong Cao, Xing-yu Xu, Jia-wei Zhang, Bo Chen, Mao-ming Xiong

**Affiliations:** 1 Anhui Medical University, Hefei, Anhui, China; 2 Department of General Surgery, The First Affiliated Hospital of Anhui Medical University, Hefei, Anhui, China; University of Kansas Medical Center, UNITED STATES

## Abstract

**Aims:**

The mammalian target of rapamycin (mTOR) and phosphorylated mTOR (p-mTOR) occurring downstream in the PI3K/Akt/mTOR pathway, are regarded as potential prognostic markers for gastric cancer (GC). However, the prognostic value of mTOR/p-mTOR expression remains controversial. In this study, we determined the expression of mTOR, p-mTOR, p70S6k, and p-p70S6K in GC, and investigated the correlation between their overexpression, clinicopathological parameters, and overall survival (OS).

**Methods:**

The expression of mTOR, p-mTOR, p70S6k, and p-p70S6K was examined in 120 GC patients by immunohistochemistry (IHC). The association of protein expression with clinicopathological features and OS was explored. The p-mTOR expression was detected in normal, adjacent, and GC tissues using Western blot. Eligible studies retrieved from PubMed, Ovid, Web of Science and Cochrane databases, were reviewed in this meta-analysis.

**Results:**

IHC showed that the rates of expression of the signal transduction molecules mTOR, p-mTOR, p70S6k and p-p70S6K in GC were 60.8%, 54.2%, 53.3% and 53.3%, respectively. Overexpression of mTOR and p70S6K showed no significant association with clinical variables. Expression of p-mTOR was significantly associated with differentiation (*P* < 0.01), depth of invasion (*P* < 0.01), lymph node metastasis (*P* = 0.04) and TNM stage (*P* = 0.02). Expression of p-p70S6K was associated with differentiation (*P =* 0.006), depth of invasion (*P* < 0.001), and TNM stage (*P* = 0.02). In survival analysis, differentiation, depth of invasion, lymph node metastasis and TNM stage were not related to OS (all *P* > 0.05). Furthermore, p-mTOR and p-p70S6K expression, but not mTOR and p70S6K, were tightly associated with OS of GC patients (*P =* 0.006 and *P* < 0.001, respectively). In Western blot, p-mTOR was significantly higher in GC tissues than in normal and adjacent tissues. In the present meta-analysis, mTOR overexpression showed no relationship with any clinicopathological variables. However, p-mTOR was correlated with depth of invasion, and TNM stage (all *P* < 0.05), and its overexpression was associated with a shorter survival time (*P* < 0.001).

**Conclusion:**

The results suggest that p-mTOR is a more valuable prognostic factor than mTOR in GC.

## Introduction

Gastric cancer (GC) is one of the most common cancers. According to the Global Cancer Statistics, 2012 [1], GC ranks sixth among all tumors in terms of the standardized incidence. About 60% of the new GC cases occur in eastern Asia [2], especially China. Tumor stage is a key factor for survival of GC patients. However, due to delayed diagnosis [3], most GC patients are at an advanced stage of cancer or distant metastasis. Despite palliative surgery, the 5–year overall survival (OS) is poor, with the median OS less than 1 year [4]. After surgical resection, the prognosis of patients with advanced GC is not ideal. Therefore, a novel prognostic biomarker for GC is necessary.

The phosphatidylinositol 3-kinase/protein kinase B/mammalian target of rapamycin (PI3K/Akt/mTOR) pathway is known to be frequently activated in several types of cancer and is essential for cancer cell survival, proliferation, angiogenesis, and resistance to chemotherapy [5–6]. Ligand binding to receptors triggers tumor growth and progression mediated via Akt, a downstream effector of PI3K pathway [7]. Moreover, mTOR, a serine/threonine protein kinase expressed in the PI3K pathway, acts as a downstream mediator in the PI3K/Akt signaling pathway [8]. It is a key regulator of eukaryotic cell growth and plays a critical role in regulating several cellular functions, including proliferation, differentiation, tumorigenesis, angiogenesis, autophagy, and apoptosis [9–10]. The mTOR activity is mediated by p-AKT. The p-mTOR expression is significantly correlated with the prognosis of gastrointestinal tumors, such as GC [11] and colorectal cancer [12], leading to decreased survival time.

Few studies have investigated the correlation between mTOR, p-mTOR, and prognostic variables comprehensively in GC. The aim of the present study was to determine the expression of mTOR and p-mTOR in GC and its correlation with clinicopathological characteristics and OS.

## Materials and Methods

### Patients and samples

GC tissue samples (120) were collected from patients who underwent total or subtotal gastrectomy at the First Affiliated Hospital of Anhui Medical University from 2010 to 2011, without receiving preoperative chemo- or radiotherapy. The patients’ age, sex, tumor location, tumor size, differentiation, depth of invasion, lymph node metastasis, distant metastasis, and TNM stage were determined by a review of their medical records. Each tumor sample was classified according to the tumor–node–metastasis (TNM) classification advocated by the International Union against Cancer [13]. Follow-up duration was determined from the date of surgical treatment until the event (death or recurrence) or censoring. The study was approved by the ethics committee of the University.

### Immunohistochemistry

The tissue samples were fixed in 10% neutral formalin and embedded in paraffin for sectioning and staining according to the manufacturer’s instructions. The 3- to 5-μm-thick tissue sections were deparaffinized and hydrated in xylene and serially diluted ethanol, respectively. The endogenous peroxidase was blocked by incubation with 3% H_2_O_2_ for 10 min. Antigen retrieval was performed in a microwave oven using citrate solution. Subsequently, the tissue sections were incubated with the appropriate primary antibody for 12 h to 16 h at 4°C. The slides were washed three times in phosphate-buffered saline (PBS) and incubated with secondary antibody for 20 min. After three further washes in PBS, diaminobenzidine tetrahydrochloride (DAB) was used before counter staining with hematoxylin. The following primary antibodies were used: mTOR (1:150), p-mTOR (1:200) (Abcam, Cambridge, UK), p70S6K (1:150) (Abcam, Cambridge, UK) and p-p70S6K (1:100) (Elabscience, Wuhan, China).

### Evaluation of immunohistochemistry

The results of immunohistochemical staining for mTOR and p-mTOR were evaluated by two independent investigators according to a semiquantitative grading system based on the proportion of stained cells and their intensity [14]. The results of immunohistochemical staining were also evaluated under similar scoring criteria. Staining intensity was scored as: 0 (negative), 1 (weak), 2 (moderate), or 3 (strong). The percentage of positive epithelial cells was scored as: 0 (no staining), 1(<1/3 staining), 2 (1/3 to 2/3 staining), and 3 (>2/3 staining). A histological score was generated as the product of intensity and the percentage of positive epithelial cells. The results of immunostaining were divided into two groups, with 0–2 score regarded as negative and >2 score considered as positive expression.

### Protein extraction and Western blot

Total protein was extracted from the normal mucosa, para-carcinoma and corresponding tumor tissues of 10 GC patients using RIPA lysis buffer (Beyotime, Shanghai, China). The protein concentration was quantified using the Enhanced BCA Protein Assay Kit. The equivalent proteins in each pair of specimens were separated by SDS–PAGE on 12% polyacrylamide gels and electrotransferred to polyvinylidene fluoride membranes. After blocking in TBST (Tris-buffered saline/Tween-20 buffer) containing 5% skim milk for 1 h at room temperature, the membrane was incubated in TBST solution containing anti-p-mTOR (1: 2000, Abcam, Cambridge, UK) overnight at 4°C. After washing three times in TBST, the membranes were incubated with the corresponding secondary antibodies in TBST along with 3% skim milk powder for 1 h at room temperature. After three washing steps in TBST, the band intensity was measured using the BandScan software. The p-mTOR band intensities were normalized to GAPDH signals.

### Statistical analysis

Statistical analyses were performed using the SPSS version 16.0 (SPSS Inc., Chicago, IL, USA). Associations between variables were examined using Pearson’s chi-squareand, Continuity correction Fisher’s exact tests. Survival curves were calculated by the Kaplan–Meier method, and the statistical significance was evaluated using the log-rank test. Results were considered statistically significant if *P*<0.05.

## Meta-Analysis

### Aims

Because of a small number of patients, a meta-analysis was conducted to confirm the previous results of IHC and survival analysis, and fully investigate whether exist relationship between mTOR, p-mTOR and clinicopathological parameters, OS through meta-analysis.

### Methods

**Fig 1:**
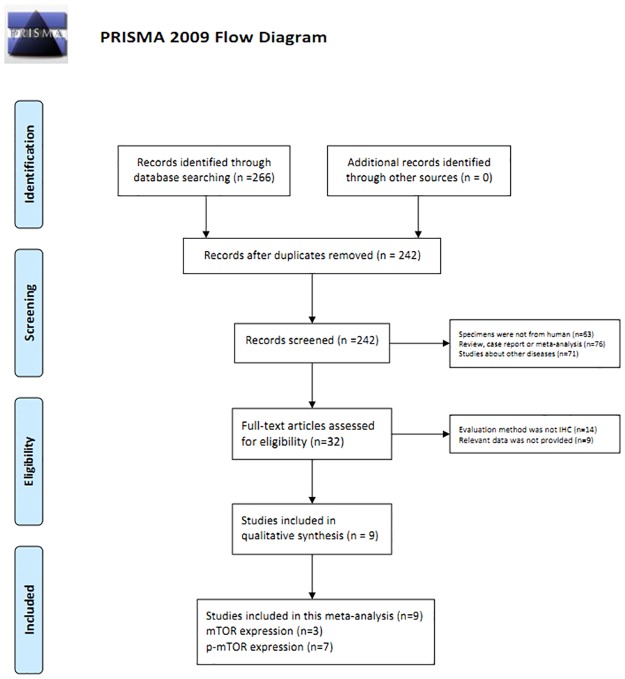
Flow diagram of study selection procedure.

Relevant articles were searched from PubMed, Ovid, Web of Science, and Cochrane from Feb 2002 to Jul 2016. The search terms used were: ("mTOR" OR "the mammmalian target of Rapamycin" OR "p-mTOR" OR "phosphrylated mTOR" OR "phosphorylated mammmalian target of Rapamycin") AND ("gastric" OR "stomach" OR "cardia" OR "gastrointestinal") AND ("adenocarcinoma" OR "carcinoma" OR "cancer" OR "tumour" OR "neoplasm" OR "tumor"). The full texts of the studies were reviewed to determine eligibility based on the inclusion criteria (Fig 1).

The full texts of the studies were read and selected if they met the following inclusion criteria: (1) GC was identified, (2) expression of proteins was evaluated by IHC, (3) clinicopathological parameters and OS were available, (4) standards to assess the protein expression was consistent across different studies, and (5) the article was published in English and Chinese. Two investigators (Guo-dong Cao and Xing-yu Xu) extracted the data independently based on consensus. The following data were extracted: first author’s name, year of publication, total number of patients, clinicopathological parameters, and survival time. During the data extraction, any disagreements were resolved by a third investigator (Bo Chen) and a consensus was reached. Two investigators (Guo-dong Cao and Xing-yu Xu) assessed the quality of included studies using the Newcastle–Ottawa scale [15].

All the statistical analyses were performed using the STATA software (version 11.0, StataCorp LP, College Station, TX, USA). The crude odds ratio (OR) and 95% confidence interval (CI) were used to estimate the strength of the association between mTOR, p-mTOR and clinicopathological parameters of GC patients. Risk ratios (RR) and 95% CIs were used in this meta-analysis to estimate the association of the status of pathway-related proteins with OS. *I*^2^ value, which indicated the percentage of total variation across studies, was used to assess statistical heterogeneity. Random-effects models (*I*^2^ >50% or *P* < 0.1) of analysis were used if significant heterogeneity was detected. Otherwise, fixed-effects models were used. Begg's rank correlation and Egger's weighted regression methods were used to determine potential publication bias (*P* < 0.05 indicates statistically significant publication bias).

## Results

### Expression of mTOR, p-mTOR, p70S6k and p-p70S6K in GC

**Fig 2:**
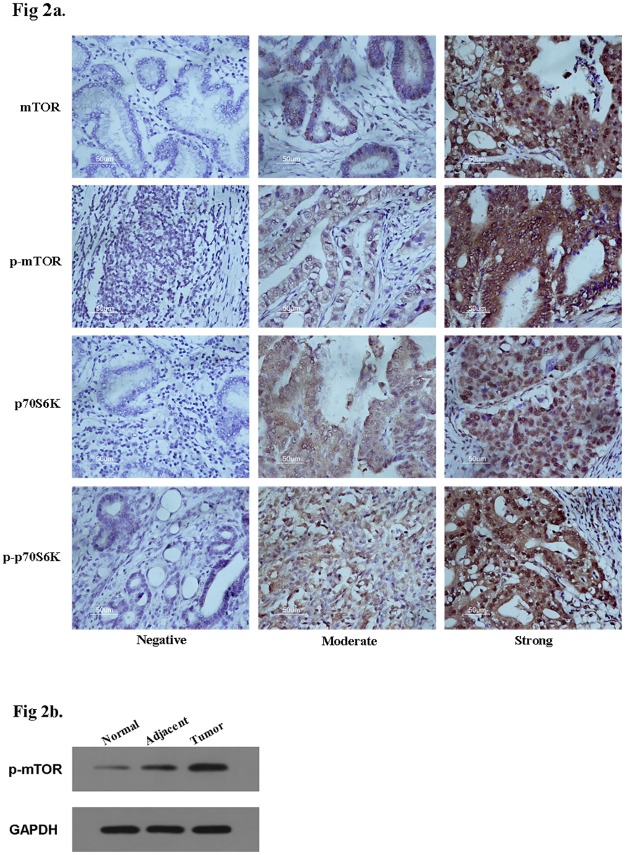
**A,** Immunohistochemical (IHC) staining of mTOR, p-mTOR, p70S6K and p-p70S6K expression in gastric cancer**. B,** Western Blot analysis of p-mTOR expression in gastric tissues.

The IHC images were demonstrated in Figure 2a (Fig 2a). According to the IHC results, mTOR and p-mTOR were highly expressed in GC tissue (Table 1). The overall rate of mTOR overexpression in 120 GC patients was 60.8%, and the overall rate of p-mTOR overexpression in 120 GC patients was 54.2%. IHC showed that the concurrent rate of p70S6k and p-p70S6K expression in GC was 53.3%. No significant difference was found between this study and previous studies (Table 2). In addition, according to Spearman correlation analysis, mTOR and p-mTOR were both significantly linked to p-p70S6K expression (*P* = 0.002 and *P* < 0.001, respectively, Table 3).

**Table 1:** Association between the clinicopathological parameters and mTOR, p-mTOR expression in 120 cases of gastric cancer.

Variable		Total patients	mTOR-positive	mTOR-negative	*P* value	p-mTOR-positive	p-mTOR-negative	*P* value
Sex	Male	80	50	30	0.60	46	34	0.30
	Female	40	23	17		19	21	
Age	<60y	49	21	28	**<0.01**	26	23	0.84
	>60y	71	52	19		39	32	
Tumor size	<3cm	15	9	6	0.58	9	6	0.63
	>3cm	105	64	41		56	49	
Differentiation	Well/moderate	102	60	42	0.28	49	53	**<0.01**
	Poor	18	13	5		16	2	
Tumor location	Upper/Medium	77	49	28	0.40	43	34	0.62
	Low	43	24	19		22	21	
Depth of invasion	T1+T2	22	10	12	0.10	6	16	**<0.01**
	T3+T4	98	63	35		59	39	
LN metastasis	N0	25	12	13	0.14	9	16	**0.04**
	N1+N2+N3	95	61	34		56	39	
Metastasis	M0	114	70	44	0.58	63	51	0.53
	M1	6	3	3		2	4	
TNM stage	I+II	33	15	18	0.03	12	21	**0.02**
	III+IV	87	58	29		53	34	

LN metastasis: lymph node metastasis. TNM stages are based on tumor-node-metastasis classification advocated by International Union against Cancer.

**Table 2:** Association between the clinicopathological parameters and p70S6K, p-p70S6K expression in 120 cases of gastric cancer.

Variable		Total patients	p70S6K-positive	p70S6K-negative	*P* value	p-p70S6K-positive	p-p70S6K-negative	*P* value
Sex	Male	80	45	35	0.37	43	37	0.90
	Female	40	19	21		21	19	
Age	<60y	49	21	28	0.06	24	25	0.43
	>60y	71	43	28		40	31	
Tumor size	<3cm	15	6	9	0.27	7	8	0.58
	>3cm	105	58	47		57	48	
Differentiation	Well/moderate	102	55	47	0.76	49	53	**0.006**
	Poor	18	9	9		15	3	
Tumor location	Upper/Medium	77	40	37	0.68	41	36	0.98
	Low	43	24	19		23	20	
Depth of invasion	T1+T2	22	7	15	**0.03**	4	18	**<0.001**
	T3+T4	98	57	41		60	38	
LN metastasis	N0	25	15	10	0.45	11	14	0.293
	N1+N2+N3	95	49	46		53	42	
Metastasis	M0	114	62	52	0.42	61	53	1.00
	M1	6	2	4		3	3	
TNM stage	I+II	33	15	18	0.29	12	21	**0.02**
	III+IV	87	49	38		52	35	

LN metastasis: lymph node metastasis. TNM stages are based on tumor-node-metastasis classification advocated by International Union against Cancer.

**Table 3:** Spearman correlation analysis between mTOR/p-mTOR and p70S6K/p-p70S6K.

	mTOR		p-mTOR	
	Spearman correlation	*P* value	Spearman correlation	*P* value
p70S6K	0.023	0.802	0.029	0.751
p-p70S6K	0.276	**0.002**	0.481	**<0.001**

As one of the major downstream proteins of PI3K/Akt/mTOR pathway, the activation of mTOR was detected in 10 paired GC samples including adjacent and normal tissues by Western blot. The p-mTOR expression was significantly higher in GC tissues indicating that it was probably related to GC tumorigenesis (Fig 2b).

### Association between mTOR, p-mTOR, p70S6k, and p-p70S6K expression and relevant parameters

As shown in Tables 1 and 2, the overexpression of mTOR was not significantly associated with sex, tumor size, differentiation, and tumor location, depth of invasion, and lymph node metastasis, or distant metastasis. However, mTOR expression was significant correlated with TNM stage (*P* = 0.03). The p70S6K expression showed no significant association with any other clinical parameters, except depth of invasion (*P* = 0.03). Meanwhile, in the present study, p-mTOR was significantly linked to differentiation (*P* < 0.01), depth of invasion (*P* < 0.01), lymph node metastasis (*P* = 0.04) and TNM stage (*P* = 0.02). The p-p70S6K expression was associated with differentiation (*P =* 0.006), depth of invasion (*P* < 0.001), and TNM stage (*P* = 0.02). The positive expression of p-mTOR and p-p70S6K suggested the possibility of poor differentiation, deeper invasion, positive lymph node metastasis and delayed TNM stage.

### Survival analysis

**Fig 3:**
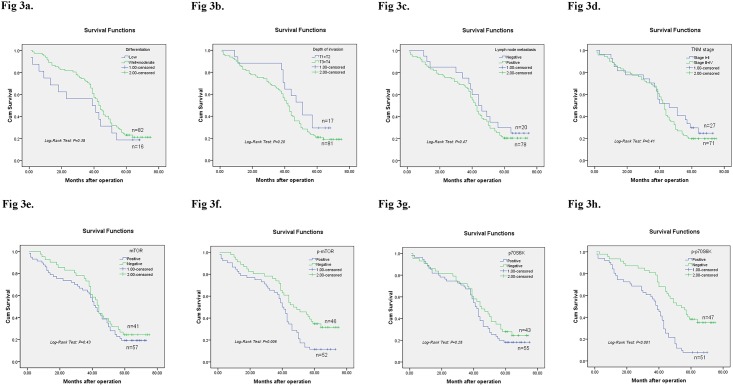
Kaplan-Meier survival curves for overall survival of 98 gastric cancer patients. Differentiation (3a), depth of invasion (3b), lymph node metastasis (3c), TNM stage (3d), mTOR (3e) and p70S6K (3g) has no relationship with overall survival rate, all *P*>0.05 analyzed by Log-Rank test. While the most interesting phenomenon is that p-mTOR (3f) and p-p70S6K (3h) overexpression are significantly associated with overall survival rate of GC patients, however, mTOR has no relationship with overall survival.

Survival analysis was demonstrated in Fig 2. OS was not related to differentiation (Log-Rank test: *P* = 0.38, Fig 3a), depth of invasion (Log-Rank test: *P* = 0.20, Fig 3b), lymph node metastasis (Log-Rank test: *P* = 0.47, Fig 3c) or TNM stage (Log-Rank test: *P* = 0.41, Fig 3d) in GC patients. As shown in Fig 3e, no association was found between mTOR and OS (Log-Rank test: *P* = 0.43). GC patients with p-mTOR overexpression showed significantly shorter overall survival rates than p-mTOR negative GC patients (Log-Rank test: *P* = 0.006) (Fig 3f). Further, p-p70S6K expression was significantly associated with OS of GC patients (Log-Rank test: *P <* 0.001) (Fig 3g). However, no such association was found between OS and p70S6K (Log-Rank test: *P =* 0.28) (Fig 3h).

### Univariate and multivariate analysis of OS

The prognostic relevance of mTOR, p-mTOR and several clinical factors was evaluated using univariate and multivariate analysis, respectively. In univariate analysis, none of the clinical factors or mTOR was associated with OS, except for p-mTOR (Hazard ratio = 1.88, 95% CI: 1.19–2.99, *P* = 0.01, Table 4) and p-p70S6K (Hazard ratio = 2.64, 95% CI: 1.65–4.23, *P <* 0.001, Table 2). The multivariate Cox proportional hazards model showed a significant correlation between p-p70S6K expression and OS (Hazard ratio = 3.00, 95% CI: 1.45–6.20, *P* = 0.003, Table 2).

**Table 4:** Univariate analysis and multivariate analysis of overall survival in 120 gastric cancer patients.

Variable	Univariate analysis			Multivariate analysis		
	Hazard Ratio	95% CI	*P* value	Hazard Ratio	95% CI	*P* value
Sex	1.15	0.70–1.89	0.58	1.14	0.68–1.91	0.62
Age	0.93	0.59–1.46	0.75	0.70	0.41–1.91	0.19
Tumor size	0.94	0.47–1.90	0.87	0.75	0.34–1.64	0.47
Differentiation	1.30	0.72–2.37	0.38	0.86	0.43–1.70	0.66
Tumor location	1.09	0.68–1.75	0.71	1.03	0.60–1.76	0.93
Depth of invasion	1.49	0.80–2.76	0.21	1.20	0.51–2.83	0.67
LN metastasis	1.31	0.73–2.34	0.36	1.11	0.53–2.30	0.79
Metastasis	0.83	0.26–2.63	0.75	1.29	0.33–5.10	0.72
TNM stage	1.30	0.77–2.19	0.33	0.90	0.42–1.95	0.79
mTOR expression	1.33	0.84–2.11	0.22	0.88	0.53–1.48	0.64
p-mTOR expression	**1.88**	**1.19–2.99**	**0.01**	1.14	0.60–2.18	0.70
p70S6K expression	1.28	0.81–2.02	0.29	0.94	0.56–1.59	0.82
p-p70S6K expression	**2.64**	**1.65–4.23**	**<0.001**	**3.00**	**1.45–6.20**	**0.003**

LN metastasis: lymph node metastasis

### Study characteristics of meta-analysis

After reviewing the abstracts and full texts, three studies of mTOR [16–18] and seven studies involving p-mTOR [11, 18–23] overexpression in GC met the inclusion criteria and were selected, respectively (Fig 3). The characteristics of the eligible studies are listed in Table 5. The study samples were analyzed using IHC and the standards for assessment were almost consistent. The mTOR positive expression in the eligible studies ranged from 51.5% to 54.6%, and the total rate of mTOR overexpression in GC patients of all the studies was 53.9% (817/1517). The rate of p-mTOR overexpression in GC ranged from 31.6% to 74.0%, and the overall rate was 48.3% (1387/2874).

**Table 5:** Clinicopathological parameters and quality scores of sutides comparing mTOR/p-mTOR positive GC with mTOR/p-mTOR negative GC.

Protein	Study	Year	Number of Patient	Sex		Age		Differentiation		Depth of invasion		LN metastasis		Metastasis		Tumor stage		Quality score
				male	female	<60	>60	Well	Poor	T1+T2	T3+T4	Positive	Negative	Positive	Negative	I+II	III+IV	
				mTOR(+)	mTOR(-)	mTOR(+)	mTOR(-)	mTOR(+)	mTOR(-)	mTOR(+)	mTOR(-)	mTOR(+)	mTOR(-)	mTOR(+)	mTOR(-)	mTOR(+)	mTOR(-)	
mTOR	This study	2016	120(73*vs*.47)	50/23	30/17	21/52	28/19	60/13	42/5	10/63	12/35	61/12	34/13	3/70	3/44	15/58	18/29	NA
	Li	2012	33(17*vs*.16)	8/9	7/9	NA		4/13	11/5	NA		13/4	3/13	NA		3/14	15/1	7
	Yu	2009	1072(545*vs*.527)	395/150	362/165	266/279	271/256	370/175	264/263	175/370	137/390	185/360	171/356	NA		224/321	203/324	9
	Xiao	2009	412(255*vs*.157)	179/76	109/48	NA		NA		143/112	79/78	173/82	90/67	NA		NA		8
p-mTOR	This study	2016	120(65*vs*.55)	46/19	34/21	26/39	23/32	49/16	53/2	6/59	16/39	56/9	39/16	2/63	4/51	12/53	21/34	NA
	Xu 2010	2010	181(93*vs*.88)	66/27	63/25	45/48	50/38	30/63	29/59	22/71	30/58	78/15	59/29	NA		28/65	45/43	8
	Bian 2015	2015	396(293*vs*.103)	196/97	65/38	121/172	56/47	NA		88/205	44/59	187/106	45/58	NA		133/160	66/37	7
	Inokuchi	2011	126(81*vs*.45)	57/24	31/14	52/29	33/12	NA		NA		49/32	18/27	28/53	10/35	35/46	28/17	7
	Yu	2009	1072(499*vs*.573)	345/154	412/161	217/282	320/253	307/192	327/246	135/346	177/396	132/367	224/349	NA		172/327	255/318	9
	An	2010	290(131*vs*.159)	88/43	97/62	NA		52/79	43/116	NA		35/95	61/98	NA		NA		9
	Murayama	2009	109(69*vs*.40)	51/18	26/14	NA		NA		NA		40/29	13/27	NA		NA		8
	Byeon	2014	700(221*vs*.479)	158/63	318/161	110/111	258/221	NA		NA		NA	NA	NA		116/105	259/220	7

GC: gastric cancer; NA: not available. TNM stages are based on tumor-node-metastasis classification advocated by International Union against Cancer. Quality score: use the Newcastle-Ottawa scale (stars)

### Correlation of mTOR and p-mTOR with clinicopathological parameters

As shown in Table 6, no correlation was found between mTOR overexpression and any clinicopathological parameters, such as differentiation (OR = 1.59, 95%CI: 0.33–07.57, *P* = 0.56), depth of invasion (OR = 0.88, 95%CI: 0.59–1.31, *P* = 0.54), lymph node metastasis (OR = 1.72, 95%CI: 0.98–3.01, *P* = 0.06) or TNM stage (OR = 3.13, 95%CI: 0.72–13.61, *P* = 0.13).

**Table 6:** Meta-analysis of a putative association between clinicopathological parameters and mTOR, p-mTOR expression in GC.

Protein	Parameters	Number of studies	Number of patients	Heterogeneity		Model	OR(95%CI)	*P* value
				*I*^2^(%)	*P* value			
mTOR	Sex (male/female)	4	1637	0	0.95	FE	1.16(0.94,1.43)	0.17
	Age (>60/<60)	2	1192	88	0.004	RE	1.90(0.59,6.06)	0.28
	Differentiation (poor/well)	3	1225	88	0	RE	1.59(0.33,7.57)	0.56
	Depth of invasion (T3+T4/T1+T2)	3	1604	57	0.10	RE	0.88(0.59,1.31)	0.54
	LN (positive/negative)	4	1637	74	0.01	RE	1.72(0.98,3.01)	0.06
	Metastasis (positive/negative)	1	98	–	–	–	0.63(0.12,3.25)	0.58
	Tumor stage (III+IV/I+II)	3	1225	89	0	RE	3.13(0.72,13.61)	0.13
p-mTOR	Sex (male/female)	8	2994	0	0.60	FE	1.09(0.93,1.28)	0.30
	Age (>60/<60)	5	2469	0	0.44	FE	1.46(1.24,1.72)	**<0.001**
	Differentiation (poor/well)	4	1663	75	0.01	RE	0.99(0.57,1.72)	0.87
	Depth of invasion (T3+T4/T1+T2)	4	1751	54	0.06	RE	1.63(1.08,2.45)	**0.02**
	LN (positive/negative)	7	2294	90	0	RE	1.57(0.83,2.98)	0.17
	Metastasis (positive/negative)	2	246	58	0.12	RE	1.05(0.25,4.44)	0.94
	Tumor stage (III+IV/I+II)	6	2595	58	0.04	RE	1.73(1.29,2.32)	**<0.001**

OR: odds ratio; CI: confidence interval; FE: fixed-effect model; RE: random-effect model; LN metastasis: lymph node metastasis

**Fig 4:**
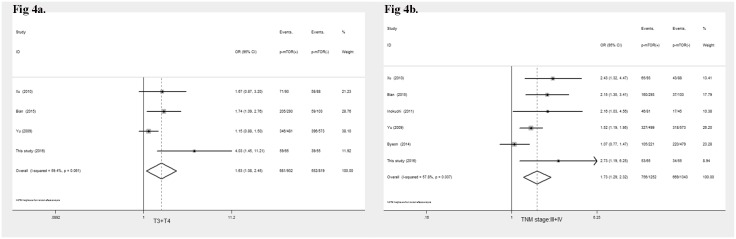
Forrest plot of odds ratio for the association of p-mTOR overexpression and lymph node metastasis (4a), TNM stage (4b).

The p-mTOR overexpression was strongly associated with depth of invasion (OR = 1.63, 95%CI: 1.08–2.45, *P* = 0.02, Fig 4a), and tumor stage (OR = 1.73, 95%CI: 1.29–2.32, *P* < 0.001, Fig 4b). The p-mTOR overexpression was independent of sex, age, differentiation, lymph node metastasis, and distant metastasis suggesting that p-mTOR was involved in tumor progression.

### Correlation of mTOR and p-mTOR overexpression with OS

**Fig 5:**
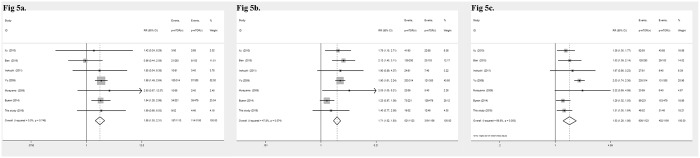
Forrest plot of the risk ratio for the association of p-mTOR and OS. (5a) Association between p-mTOR overexpression and 1-year OS. (5b) Association between p-mTOR overexpression and 3-year OS. (5c) Association between p-mTOR overexpression and 5-year OS.

The survival time was extracted from the Kaplan–Meier survival curves analyzed by Enguage Digitizer software. In the present study, mTOR-positive expression was not correlated with the 1-, 3- and 5-year overall survival rate of the GC patients (Table 7). However, p-mTOR expression was significantly related to 1- (RR = 1.86, 95%CI: 1.50–2.31, *P* < 0.001, Fig 5a), 3- (RR = 1.71, 95%CI: 1.52–1.93, *P <* 0.001, Fig 5b), and 5-year (RR = 1.53, 95%CI: 1.26–1.86, *P* < 0.001, Fig 5c) OS in GC patients.

**Table 7:** Meta-analysis of a putative association between OS and mTOR, p-mTOR expression in GC.

Protein	OS	Number of studies	Number of patients	Heterogeneity		Model	OR(95%CI)	*P* value
				*I*^2^(%)	*P* value			
mTOR	1-year OS	3	1179	90	0	RE	1.02(0.38,1.2.73)	0.97
	3-year OS	3	1179	89	0	RE	1.06(0.62,1.81)	0.82
	5-year OS	3	1179	94	0	RE	1.02(0.65,1.61)	0.94
p-mTOR	1-year OS	7	2269	0	0.75	FE	1.86(1.50,2.31)	**<0.001**
	3-year OS	7	2269	48	0.07	FE	1.71(1.52,1.93)	**<0.001**
	5-year OS	7	2269	70	0.003	RE	1.53(1.26,1.86)	**<0.001**

OS: overall survival; RR: risk ratio; CI: confidence interval; FE: fixed-effect model; RE: random-effect model

### Sensitivity analysis and publication bias

**Fig 6:**
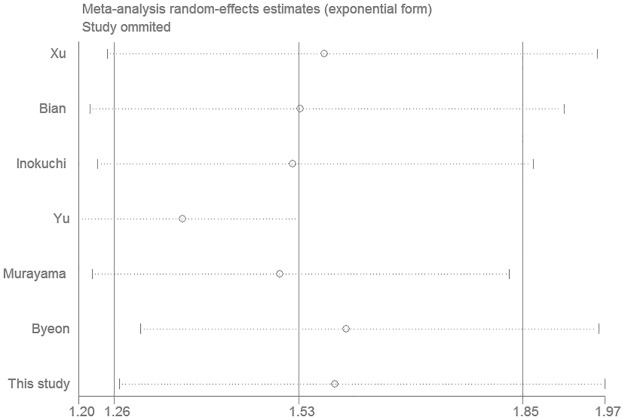
Effect of individual studies on pooled risk ratios (RR) for p-mTOR expression and overall survival (OS) in patients with gastric cancer.

In order to test the robustness of the RR estimates of OS, sensitivity analysis was conducted by individually excluding the studies and analyzing the effects on the remaining studies. As shown in Fig 6, the results of RR estimates were relatively reliable and credible as no point estimate of the omitted individual study was outside the 95% CI.

**Fig 7:**
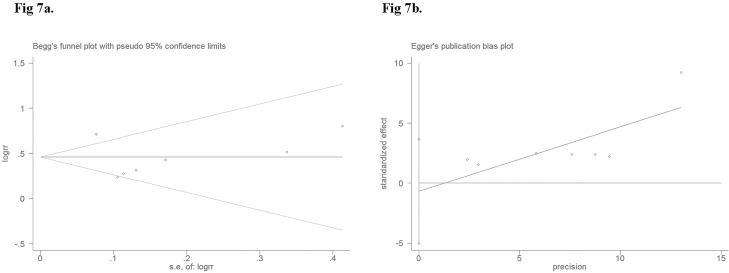
Begg’s funnel plot (7a) (*P* = 0.23) and Egger’s funnel plot (7b) (*P* = 0.70) for possible publication bias test of this study. There was no publication bias and the results are credible.

Begg's rank correlation and Egger's weighted regression methods were used to statistically assess the publication bias. As shown in Fig 7a and 7b, neither Begg’s (*P* = 0.23) nor Egger’s (*P* = 0.70) test provided any clear evidence of publication bias. These results indicate the absence of publication bias in the current study. We believe that the results of our meta-analysis are credible.

## Discussion

The prognostic role of mTOR and p-mTOR expression has been studied extensively in other types of cancers, despite controversial results. Li *et al*. [24] first investigated the prognostic value of mTOR and p-mTOR in non-small cell lung (NSCLC) cancer comprehensively in a meta-analysis. In this study, no statistically significant association was found between mTOR and p-mTOR expression, and prognosis of NSCLC patients. The mTOR is a down-stream effector of PI3K-Akt signaling pathway and is regarded as a Ser/Thr protein kinase that mediates nutrient-dependent intracellular signaling related to cell growth, proliferation, and differentiation. Previous studies have identified mTOR signaling as a potential target for anticancer therapy using several cancer models [25]. Furthermore, the mTOR is activated and phosphorylated (p-mTOR). Osaki *et al*. [26] reported that p-mTOR is frequently expressed in ovarian cancer and may be targeted to disrupt ovarian tumor cell growth. Association between mTOR and p-mTOR expression, and GC remains unclear. The role of mTOR and p-mTOR as prognostic predictors is controversial and warrants further investigation.

Interestingly, our original study demonstrated that both mTOR and p-mTOR were associated with several clinicopathological parameters such as TNM stage (*P* < 0.05). However, in the survival analysis, p-mTOR overexpression was significantly related to overall survival of GC patients (*P* = 0.006). No obvious difference was found between mTOR overexpression and OS in GC.

These inconsistent but interesting results require comprehensive investigation. Concurrently, because of limitations associated with small sample size, the meta-analysis was conducted to determine the prognostic value of mTOR and p-mTOR. Finally, in this meta-analysis, mTOR was not correlated with clinicopathological variables or OS. However, the correlation was found between depth of invasion and TNM stage, which are tumor predictors. Nonetheless, p-mTOR overexpression always indicates a shorter survival time in GC.

The mTOR has two main downstream factors, eukaryotic initiation factor 4E binding protein 1(4E-BP1) and ribosomal S6 kinase (p70S6K), which were mediated by phosphorylated mTOR activity [27]. Dephosphorylated 4E-BP1 binds to eukaryotic initiation factor 4E (eIF-4E), leading to inhibition of translation initiation. Phosphorylation of 4E-BP1 by p-mTOR releases 4E-BP1 from the mRNA cap-binding protein eIF4E, triggering the process of translation and protein synthesis [28, 29]. The p-mTOR activates p70S6K, and phosphorylated p70S6K combines with translation initiation complexes, to improve the efficiency of mRNA translation [30]. In general, p-mTOR induces phosphorylation of 4E-BP1 and P70S6K, and initiation of translation and protein synthesis. Yu *et al*. [18] reported that p-mTOR overexpression was related to clinicopathological variables and p-mTOR appears to be a more sensitive biomarker than total mTOR in predicting patient survival. Ji *et al*. [31] reported that targeting the expression of p-mTOR with specific siRNA reduced the growth and overall survival rate of Hela cervical cancer cells *in vitro*. The process of mTOR phosphorylation is suppressed by mTOR-specific inhibitors, such as everolimus and rapamycin. Phase II studies showed that treatment strategies using everolimus showed high efficacy and safety in GC, and therefore a global phase III study is currently underway [32]. Riquelme *et al*. [33] found that rapamycin treatment did not significantly alter the protein expression of total mTOR in several types of cell lines. However, rapamycin significantly decreased the phosphorylation of both mTOR and its downstream effectors, such as p70S6K1 and 4E-BP1. In a previous study [34], Yang and his co-workers used Western blot and reverse transcription polymerase chain reaction (RT-PCR) to assess the expression of mTOR in GC. The findings suggest that the expression of mTOR at the protein level was significantly lower than in the corresponding normal gastric mucosa, while the ratio of p-mTOR was significantly increased in tumor tissues. The conclusion was that the mTOR signaling pathway was activated in GC, mainly via increased mTOR phosphorylation rather than overexpression of dephosphorylated mTOR. In our original survival analysis, the results indicated that p-mTOR overexpression was significantly associated with overall survival rate. However, the mTOR expression and TNM stage were meaningless. Furthermore, in this meta-analysis, we arrived at a similar conclusion suggesting that p-mTOR plays a key role in GC as a promising predictor.

However, the limitations of the current study include: (1) small number of patients; (2) exclusion of a few eligible non-English and non-Chinese studies; and (3) insufficient number of articles. However, several advantages were as follows: (1) This is the first available study and meta-analysis of the association between mTOR, p-mTOR overexpression and clinicopathological parameters in GC. (2) We also compared the prognostic value of mTOR and p-mTOR in GC for the first time. (3) The results of this study provide theoretical support of rapamycin treatment in GC.

In summary, it is not possible to accurately predict the prognosis of GC patients on the basis of the current staging system alone. The expression of p-mTOR is significantly associated with clinicopathological parameters and OS, and plays a vital role in the progression of GC. It is a more valuable prognostic factor than mTOR in GC and may be regarded as a new predictor.

## Supporting Information

S1 File PRISMA Checklist. PRISMA 2009 checklist.
(DOC)

S2 File Ethics Certification. (DOCX)
