# Supplementary material for: Phosphorylated Mammalian Target of Rapamycin p-mTOR Is a Favorable Prognostic Factor than mTOR in Gastric Cancer
Source: PLoS One. 2016 Dec 22;11(12):e0168085. doi: 10.1371/journal.pone.0168085 (PMC5179011; doi:10.1371/journal.pone.0168085)
Supplement: S2 File — (DOCX) [file pone.0168085.s002.docx]

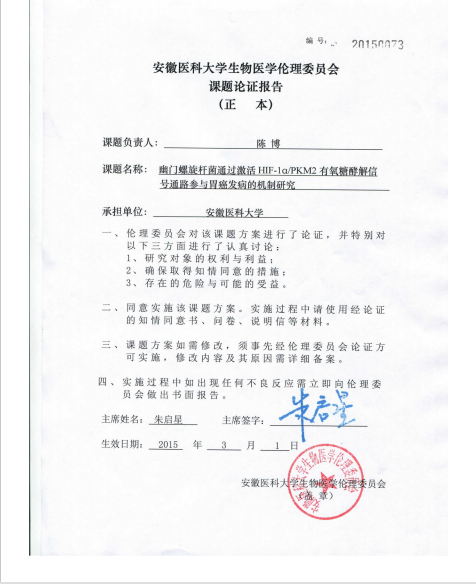


**(English Translation)**

**Approved Code:20150073**

**ANHUI MEDICAL UNIVERSITY**

**Verification of Biomedical Research Ethics Committee**

**(Formal Version)**

**Principal Investigator: Bo Chen**

**Project Name:** The molecular mechanism of enhanced activity of HIF-1α/PKM2 signaling pathway in Helicobacter pylori-associated gastric carcinogenesis

**Institution of Applicant: Anhui Medical University**

1. The bio-medical ethics committee have demonstrated the program of this project, and cautiously discussed the following three points:

①: The rights and interests of the research objects;

②: The measures to obtain the right of knowing and consent;

③: The potential danger and possible benefit.

1. Approved to perform this research plan. In this project implementation process, informed consent form, questionnaire and relevant instruction should be used.

3. If the project program needs to be revised, it must be demonstrated by the bio-medical ethics committee in advance. At the same time, the modification details and reasons should be recorded.

4. If any adverse reactions appeared, submit a written report to the bio-medical ethics committee.

The Chairman’s Name: Qi-xing Zhu Signature:

Effective Date: 2015-03-01

Biomedical Research Ethics Committee of Anhui Medical University
